# Non-invasive aerosol delivery and transport of gold nanoparticles to the brain

**DOI:** 10.1038/srep44718

**Published:** 2017-03-16

**Authors:** Ramesh Raliya, Debajit Saha, Tandeep S. Chadha, Baranidharan Raman, Pratim Biswas

**Affiliations:** 1 School of Engineering and Applied Science, Washington University in St Louis, St Louis, Missouri 63130, USA; 2 Systems Neuroscience and Neuromorphic Engineering Laboratory, Department of Biomedical Engineering, Washington University in St Louis, St Louis, Missouri 63130, USA

## Abstract

Targeted delivery of nanoscale carriers containing packaged payloads to the central nervous system has potential use in many diagnostic and therapeutic applications. Moreover, understanding of the bio-interactions of the engineered nanoparticles used for tissue-specific delivery by non-invasive delivery approaches are also of paramount interest. Here, we have examined this issue systematically in a relatively simple invertebrate model using insects. We synthesized 5 nm, positively charged gold nanoparticles (AuNPs) and targeted their delivery using the electrospray aerosol generator. Our results revealed that after the exposure of synthesized aerosol to the insect antenna, AuNPs reached the brain within an hour. Nanoparticle accumulation in the brain increased linearly with the exposure time. Notably, electrophysiological recordings from neurons in the insect brain several hours after exposure did not show any significant alterations in their spontaneous and odor-evoked spiking properties. Taken together, our findings reveal that aerosolized delivery of nanoparticles can be an effective non-invasive approach for delivering nanoparticles to the brain, and also presents an approach to monitor the short-term nano-biointeractions.

Application of nanomaterials and nanotechnology for diagnostic and therapeutic needs have gained popularity in the last decade^1^,^2^,^3^. A variety of nanomaterials such as polymeric, lipid-based, carbon and inorganic nanoparticles have been used to target various organs for the purposes of drug delivery, bio-imaging and biosensing^1^,^4^. Tissue-specific drug delivery approaches have shown to maximize the drug efficiency at a reduced dose while minimizing side effects by restricting bio-distribution of the drug in non-specific tissues^5^,^6^,^7^. However, the brain is well protected for tissue-specific drug delivery by the blood-brain barrier (BBB), which tightly controls any substance exchanges between the central nervous system and the blood^8^,^9^,^10^,^11^. The main function of the BBB is to protect the brain from potentially harmful foreign substances. As a result, BBB also prevents delivery of therapeutic agents, restricts permeability and retention of drug molecules^12^,^13^. In the past, efforts were made to temporarily open the tight blood-brain barrier junctions using either high osmolar solutions^14^ or intracerebral injections to cross the BBB^15^. However, these invasive approaches have limitations, such as tissue damage, and uncontrolled distribution of the drug from the point of injection. To improve the drug delivery to the brain while minimizing tissue damage, attempts were also made through the nasal pathway. Once drugs permeate through the nasal epithelium, they transport to the brain along olfactory nerves^16^,^17^. Since the simplest and shortest path for airborne nanoparticles to reach the central nervous system is through the olfactory tract^18^,^19^,^20^, intranasal delivery provides the fastest route and potentially a non-invasive option to deliver therapeutic agents to target cells in the brain.

Amongst the various materials explored for drug delivery, imaging and as therapeutic agents, gold nanoparticles (AuNPs) have emerged as the material of choice in a number of studies^21^. The relative ease in synthesizing and functionalizing the AuNPs of various sizes, combined with their biocompatibility and plasmonic properties make the AuNPs an excellent candidate for several biomedical applications^4^. Moreover, it has been reported that AuNPs of certain size range (<10 nm size) could cross the blood-brain barrier making it an attractive candidate for treating various neurological diseases as well^22^.

In this study, we report a non-invasive delivery approach based on aerosolized nanoparticles exposures. We chose the invertebrate (locust; *Schistocerca americana*) olfactory pathway to test the nanoparticle transport and its physiological interactions with neurons primarily due to two reasons. First, as mentioned earlier, the olfactory pathway is one of the shortest routes to deliver nanoparticles as sensory neurons in the insect antenna project directly onto central regions in the insect brain. Second, the insect olfactory pathway is very well characterized and a popular model for neural coding and behavior^23^,^24^,^25^,^26^,^27^,^28^,^29^,^30^,^31^, which enabled us to examine not only the nanoparticle accumulation but also physiological effects these nanoparticles had on the functionality of olfactory neurons in the brain. It might be worth noting that the invertebrate olfactory system is also functionally and architecturally very similar to the mammalian olfactory system^17^,^32^. To the best of our knowledge, physiological interactions of aerosolized AuNPs with neurons in a sensory pathway have not been studied earlier. Our study presents a facile, non-invasive approach for rapid delivery and transport of AuNPs into the brain while minimally disrupting the normal physiology for a limited duration following exposure.

## Results

### Synthesis, characterization and functionalization of AuNPs

**Figure 1:**
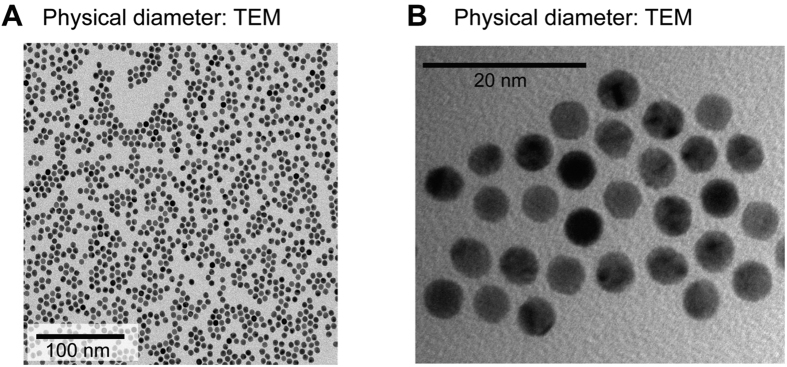
Gold nanoparticle synthesis and characterization. **(A)** A TEM image showing spherical morphology and physical diameter of AuNPs. Note that the AuNPs sizes are fairly uniform **(B)** A magnified view clearly revealing the conserved AuNPs size distribution.

AuNPs were synthesized by a seed mediated approach using gold chloride, a precursor salt^33^. Since the particle size distribution and zeta potential of nanoparticles are important factors for bio-interactions^34^,^35^, synthesized nanoparticles were customized to an average size of 5 nm in diameter and of spherical shape (Fig. 1A,B). Physical characteristics of the nanoparticles were confirmed using transmission electron microscopy (TEM). AuNPs were monodispersed (Polydispersity Index, PDI: 0.022) in water at physiological pH (7.4) and the zeta potential of AuNPs was kept at 33.2 ± 0.43 mV.

The positively charged AuNPs were partially capped by cysteine amino acid. Cysteine binds on the surface of AuNPs due to the presence of thiol (-SH) group^1^. The partial capping of AuNPs provided the control over functionalization with fluorescent dye molecules. Attached cysteine provided free amine group, which was used for binding of fluorescent dye, the fluorescein isothiocyanate (FITC) molecules^36^. The spherical shaped 5 nm gold particles have a light absorption maxima at 524 nm wavelength as shown in Fig. S1A. The absorption peak shifted to 530 nm after partial capping by cysteine, and the peak shifted further to 482 nm after FITC binding of the cysteine molecules. The fluorescent tagging of the dye molecules were also confirmed by putting a drop of as functionalized particles on a glass slide and observing it under a fluorescence microscope (Fig. S1B).

### Non-invasive - aerosol delivery of AuNPs to the locust’s brain

Following the functionalization of AuNPs, they were dispersed in water. The hydrodynamic diameter of monodispersed AuNPs at physiological pH and temperature was around 9.4 nm (Fig. S2A; PDI = 0.022). The monodispersed AuNPs were aerosolized for delivery. The size of gold nanoparticle aerosol was measured to be 13.22 ± 1.19 nm (Fig. S2B). The larger sizes of dry aerosol particle when compared to the physical diameter of the AuNPs (5 nm) might be due to the surface functionalization of the AuNPs and the formation of agglomerates.

**Figure 2:**
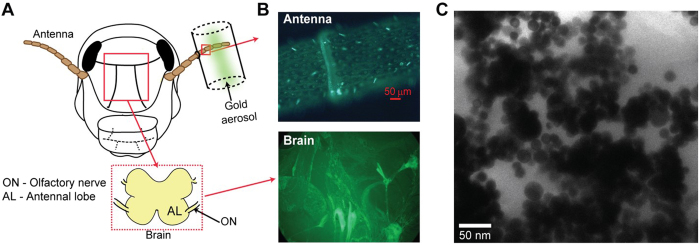
Uptake and translocation of AuNPs in the locust olfactory pathway. **(A)** A schematic of the experimental setup is shown. FITC functionalized, gold nanoparticle were aerosolized and delivered onto locust antenna. Inset reveals the details of the early invertebrate olfactory pathway. **(B)** *Top,* An image of a segment of the locust antenna after AuNP aerosol exposure is shown. Notice that sensory hairs (cone like structures on the antenna surface) show different levels of fluorescence. *Bottom*, Similar fluorescence image of a brain segment is shown. **(C)** TEM micrograph of a brain slice confirms accumulation of AuNPs in the locust brain.

Next, we delivered the functionalized AuNP aerosol to the locust antenna (Fig. 2A, Fig. S3). After 3 hours of aerosol exposure, we found that select sensory hairs in the antenna had strong FITC signals. This result suggested accumulation of fluorescent AuNPs in those sensory hairs (Fig. 2B, top). As a control, we examined the fluorescent signals from the antenna of an unexposed locust. We note that no fluorescent signals were detected in the control antenna. These results suggest that the nanoparticle uptake was inhomogeneous across different sensilla of the exposed antenna.

### Biodistribution of AuNPs in the olfactory pathway

Since these sensory neurons project directly to the brain through the olfactory nerve, we subsequently analyzed the whole brain under fluorescent microscope (Fig. 2B, *bottom*). Interestingly, we observed broad labeling of several neural processes in the locust brain indicating that the nanoparticles delivered to the insect antenna do indeed reach the brain. Finally, to further characterize the accumulation of nanoparticles in the brain, we performed sectional imaging of 75 nm thick brain slices by TEM. The micrograph, shown in Fig. 2C confirms the presence of AuNPs in a brain segment of a locust whose antenna was exposed to gold nanoparticle aerosol. Further, element “gold” was confirmed and quantified by using ICP-MS.

**Figure 3:**
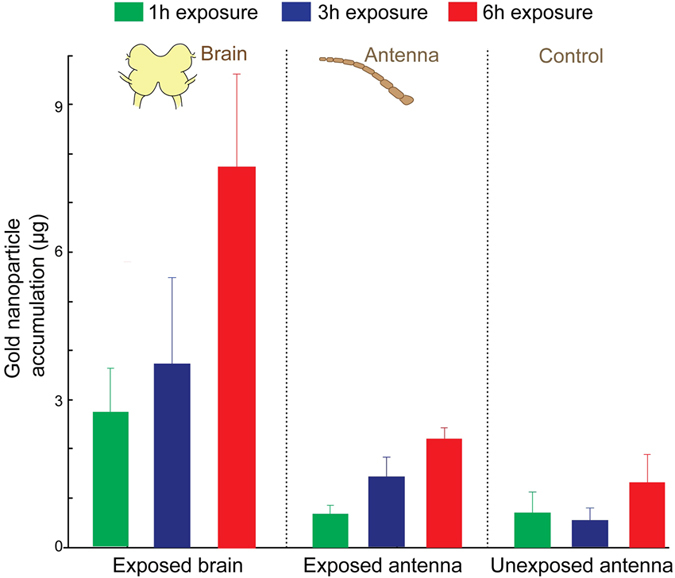
Quantification of nanoparticle transport ICP-MS analyses of insect antenna and brain following AuNP exposure are shown. Three different exposure durations were examined: 1 hour, 3 hours and 6 hours exposure. Note that the amount of nanoparticle accumulation in the exposed antenna and in the brain linearly increases with exposure duration. Accumulation of the AuNPs in unexposed antenna is shown as a control. Mean value ± S.E.M. are shown for each exposure duration. After six hours AuNPs exposure, brain accumulates 7.7 ± 1.9 μg gold, whereas, antenna accumulates 2.2 ± 0.22 μg gold, as measured by the ICP-MS.

To understand how the efficacy of the nanoparticle uptake and translocation through a sensory pathway vary with exposure durations, we subjected locusts to gold aerosol exposure of different durations. We quantified the amount of AuNPs accumulated both in the exposed antenna and in the brain using the ICP-MS. Our results indicate that the quantity of AuNPs accumulation in both the exposed antenna and the brain linearly increased with the exposure duration (Fig. 3). The locust brain, exposed with one hour of AuNP aerosol, accumulates 2.7 ± 0.8 μg, whereas, six hours exposed brain accumulates 7.7 ± 1.9 μg of nanoparticles. Similarly, one hour exposed antenna accumulated 0.6 ± 0.18 μg and six hours exposed antenna accumulated 2.2 ± 0.22 μg gold. The number concentration in the aerosol flow, to which the locust was exposed, calculated to be 2.34 × 10^5^ particles/cc. The details of the calculation is provided in the supplementary information file. The aerosol flow rate during the exposure was 1.1 L.min^−1^. The mass flow rate of the nanoparticles in the aerosol was estimated to be 0.316 μg/h. Thus the fraction of primary aerosol transported to the brain was 0.00887 per hour.

As a control, we performed the same ICP-MS analysis on the other locust antenna that was not exposed to the AuNP aerosol (Fig. 2A). In the control antenna, we did not see systematic increase in gold accumulation over time although low quantity of gold was detected. To ascertain the source of the trace amount of gold found in the control antenna, we performed ICP-MS analyses on materials that were either fed to the locusts or used during the course of experiments, such as wheat grass and soil, oats, DI water, and an acid mixture (HNO_3_ + HCl). We found a small fraction of gold in oats and in grass with soil content, which were used as locust food (Fig. S4). We surmise that this could be a potential source for low level of gold found in the control locust’s antenna/brain.

### Electrophysiology response

**Figure 4:**
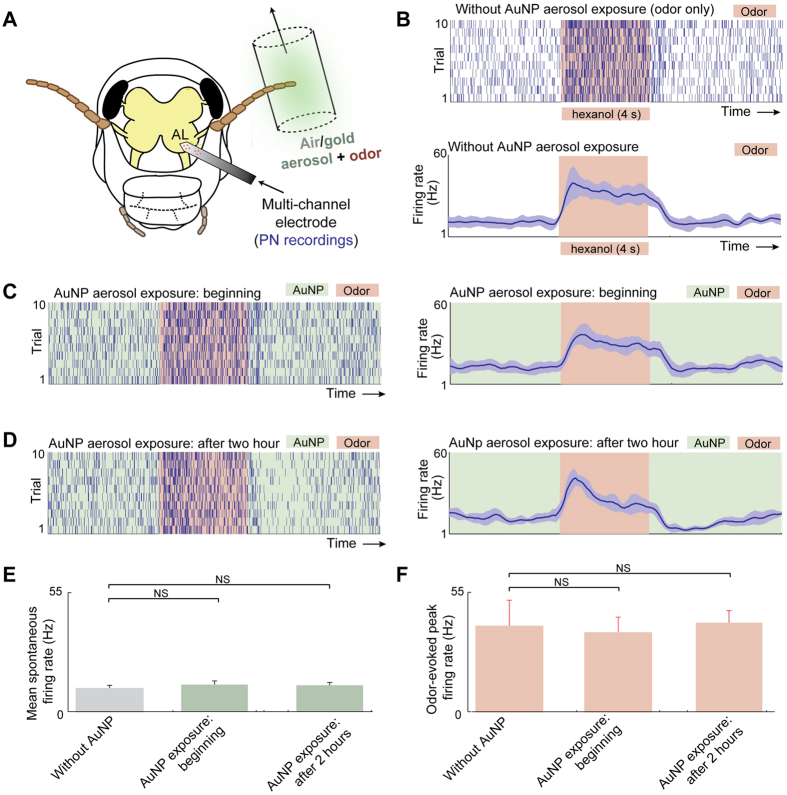
Electrophysiological characterization of bio-interactions of AuNPs. **(A)** Schematic of the odor/AuNP delivery and recording setup is shown. An odorant (hexanol 1%) was delivered atop a dry air or a gold nanoparticle aerosol background. The extracellular recording was made to monitor the responses of the principal neurons (or projection neurons; PNs) in the antennal lobe (AL). **(B)** *Top*, raster plot showing spiking activity recorded from an ensemble of neurons in the vicinity of the recording electrode. The 4 s duration of odor exposure is shown as a shaded orange box. Bottom, mean spiking activity in 100 ms time segments are shown after averaging across 10 trials. Error bars (shown in light blue) represent ± S.D. over trials. **(C)** Similar spiking and firing rate plots as in panel (**B**) but revealing responses of the same set of neurons immediately following the onset of AuNP aerosol exposure. **(D)** Similar plots as in panel (**B,C**) but now showing the population PN responses after 2 hours of continuous AuNP aerosol exposure. **(E)** A comparison of mean ± S.D of spontaneous neural firing rate (baseline spiking activity in a 5 s window without any odor exposure) is shown. Note that spike rates across three conditions are statistically comparable to each other (paired t-test, NS when *p* > 0.01, *n* = 10). **(F)** Similar analyses but comparing activity during the odor puff in all three conditions. Again the differences in spiking activities are insignificant across the three conditions (paired t-test, NS when *p* > 0.01, *n* = 10).

While the histological studies, TEM imaging, and ICP-MS results confirmed that the noninvasive delivery of AuNP aerosol onto the antenna do reach the brain, we wondered if their presence interfered with or altered the physiological responses of the olfactory neurons found in the central region. To understand this, we performed electrophysiological recordings from the insect antennal lobe (a region directly downstream to the insect antenna and analogues to the mammalian olfactory bulb). We made *in vivo* extracellular recordings from an ensemble of antennal lobe neurons using a multi-electrode array (Fig. 4A). We observed neural responses as a function of time due to odor stimulation. Spike raster shown in Fig. 4B indicate the timing of action potentials of the recorded neurons. Each row (10 s duration; one trial) reveals responses of the neurons before, during (shaded region) and after an odor puff was delivered. The average responses across trials (total spiking activity in 100 ms time segments) were computed and plotted below the spike raster plot. Note that these odor-evoked responses provided a reference response template with which comparisons could be made before and after AuNP aerosol exposures.

Subsequently, the insect antenna was exposed to the gold aerosol while the physiological responses were continuously monitored (Fig. 4C,D). As can be noted, the spiking activity immediately or 2 hours after the exposure of AuNP aerosol were qualitatively similar to those observed in the control conditions (dry air, Fig. 4B). To quantify these results, we compared the spontaneous pre-stimulus firing rates and odor-evoked spiking responses across different conditions (i.e. before and after aerosol exposure). We found that the response patterns and the firing rates of the spontaneous and the odor evoked responses remained conserved for the amount of time the physiological activity was monitored following AuNP exposures (paired t-test, non-significant, NS when *p* > 0.01, *n* = 10; Fig. 4E,F). Taken together our findings suggest that electrophysiological properties of these olfactory neurons remained unaffected for several hours following AuNP exposure.

## Discussion

Vertebrate and invertebrate olfactory systems share many similarities in their organization, functionality, neural coding strategies^17^,^32^,^37^,^38^,^39^. By employing nanoparticle directly on the antenna (equivalent to nasal epithelium in vertebrates), we investigated the functionality of projections neurons (PNs) in the insect antennal lobe which is equivalent to the mitral cells (MCs) in the vertebrate olfactory bulb. These neurons are the main output neurons (PNs or MCs) of the central circuitry (antennal lobe or olfactory bulb) and directly send their inputs to the memory and learning centers for both invertebrate and vertebrate systems.

Insect olfactory system has proven instrumental in understanding the neural code of odors and behavioral outcomes^24^,^25^,^26^,^28^,^40^,^41^,^42^,^43^,^44^,^45^. This sensory pathway also provides easy access to electrophysiological recordings from different brain centers while nanoparticle exposure 25. All of these advantages render invertebrate olfactory pathway an important target for studying the effects of nanoparticles on the brain. This study opens the door for more details individual neuron and circuit level investigations involving nanoparticle and neural functionality. The uptake and translocation rate are higher compared to a previous studies^20^,^46^,^47^. This is mainly because the previous studies (Oberdorster *et al*.^20^, or Kreyling *et al*.^46^) delivered the nanoparticles via airways through the lung in mice and relied on the nanoparticles being transported to the brain via the circulatory system crossing the blood brain barrier. It is well known that the efficiency of this delivery route is low. It is worth noting that transport of nanoparticles in gaseous phase through the nasal pathway is a topic of great interest from both environmental and medical sciences point-of-view^2^.

Recent reports have revealed similar transport and accumulation of aerosolized quantum dots^48^, ultrafine carbon particles^20^, Fullerenes^47^ and intravenously injected AuNPs^49^ to the mammalian brain. However examination of changes in physiology before and after such delivery has proven to be a challenge. Here, taking advantage of a much simpler invertebrate olfactory pathway, we have revealed that the presence of certain AuNPs may not interfere much with the physiological responses for a short-duration after exposure. Customizations of nanoparticles that are efficiently transported along the sensory pathway and at the same time have minimal interaction with the nervous system are ideal features necessary for most diagnosis and therapeutic applications in biomedical domain. We note that further studies that focus on particle mode of entry, efficacy of delivery, transport mechanisms, and long term toxicity are still needed to complement the effort presented here.

In summary, we developed techniques to systematically synthesize, characterize, and deliver customized AuNP aerosols in a controlled fashion. Our results indicate that the AuNP aerosol presented to a peripheral sensory organ (i.e. insect antenna) gets transported to the brain.

## Methods

To study the transport and accumulation of AuNPs in the brain, we custom designed the particles and exposed them by electrospray onto insect antenna. Qualitative and quantitative studies of the particles transport and accumulation were performed using different microscopy techniques (details below). Finally, bio-interaction of AuNPs was observed by multi-electrode electrophysiology. All the chemicals used in this study to synthesize AuNPs were purchased from Sigma Aldrich, St. Louis, USA, unless specified.

### Synthesis of AuNPs

Spherical AuNPs were synthesized in two steps by a seed-mediated approach using cetyl tri-methyl ammonium chloride (CTAC) as a surfactant. In the first step, seed solution was prepared by vigorous mixing (500 rpm for 1 h at 30 °C; using analog vortex mixer (Fisher Scientific, USA) of 10 mL of HAuCl_4_ (2.5 × 10–4 M) aqueous CTAC (0.1 M) solution with 0.45 mL of cold NaBH_4_ (0.02 M). Further, the seed solution was aged for 60 minutes (at 30 °C) to decompose the excess NaBH_4_. In the second step, the growth solution was prepared by adding 514 μL of HAuCl_4_ (4.86 mM), 10 μL of NaBr (0.01 M), and 90 μL of ascorbic acid (0.04 M) to 5 mL of aqueous CTAC (0.1 M) solution. Finally, 25 μL of the seed solution was added to a colorless growth solution, under vigorous agitation, and left undisturbed overnight at room temperature. To control the size of the AuNPs, the molar ratio of the ascorbic acid to the seed solution was varied.

### Functionalization of AuNPs

Synthesized AuNPs were functionalized with the fluorescent dye to observe its bio-distribution in the brain. In the present study, we used fluorescein isothiocyanate to functionalize AuNPs, as described elsewhere^50^. In brief, synthesized AuNPs were suspended in water (10 mg L^−1^) and mixed with cysteine (50:1 ratio v/v). Cysteine binds to the surface of AuNPs due to the thiol (–SH) group and has a free amino group (–NH_2_) group. The amine-terminated surface provides a platform to attach FITC to the gold nanoparticle.

### Characterization of AuNPs

The synthesized and functionalized AuNPs were characterized using a UV-vis spectrometer (Varian Inc., Cary 50) for surface enhanced Raman scattering property. The physical diameter of the AuNPs was characterized by TEM (FEI Tecnai G2 Spirit, USA), and the size distribution and zeta potential were measured offline by Dynamic Light Scattering technique (DLS; Malvern Instruments, Zetasizer Nano ZS, USA). Additionally, FITC tagged AuNPs were also imaged by fluorescence microscope (Zeiss AXIO Observer Z1, USA). Real-time characterization of AuNPs for size and number distribution was obtained by Scanning Mobility Particle Sizer (SMPS; TSI Inc., USA).

### Aerosol of AuNPs

A solution of AuNPs (5 mg/L, w/v) was aerosolized using a commercial electrospray aerosol generator (Electrospray Aerosol Generator, Model 3480, TSI Inc., USA). Before electrospraying, the conductivity of the solution was adjusted to 299 μΩ^−1^ cm^−1^ by the addition 20 mM ammonium acetate solution. A mixture of nitrogen at a flow rate of 1.0 L.min^−1^ and carbon dioxide at a flow rate of 0.1 L.min^−1^ were used as the carrier gas. A stable Taylor cone jet was obtained at 2.5 kV, and the droplets were neutralized using a Po-210 source (Fisher Scientific, USA) in a chamber. The nanoparticles were then delivered to the locust antenna (experimental set shown in Fig. S3). It is important to know the real-time exposure concentration of AuNPs and mean geometric diameter. Therefore, the aerosol size distribution and number concentration were carried out using SMPS.

### Aerosol exposure

Aerosol exposure of AuNPs was conducted using young-adult (post-fifth instar) locusts (*Schistocerca americana*) of either sex raised in a crowded colony. First, the insect was secured in a recording chamber and the antenna was stabilized using batik wax. One of the antennae was inserted into a plastic tube through a small hole (comparable to the diameter of the antenna), which was then sealed off with batik wax to ensure localized delivery and to ensure no leakage of the AuNP aerosols outside the delivery system. This plastic tube was then connected to the electrospray system. In this study, three different aerosol exposure durations were tested. Right after the exposure, both antennae, and the brain were excised to determine whether there was an uptake of the gold nanoparticles and the amount of the AuNPs accumulated. Multiple locusts were tested for each exposure condition.

### Electrophysiology

To study the bio-interaction properties of translocated AuNPs, electrophysiological recordings were conducted from the neurons in the antennal lobe (a sensory neural circuit directly downstream to the insect anntenna). Locusts were immobilized in a recording chamber with both antennae intact. Next, following surgical procedure described in earlier works, the brain was exposed, desheathed, and superfused with locust saline^25^. One of the antennae was inserted into the carrier gasstream. A mixture of nitrogen at a flow rate of 1.0 L.min^−1^ and carbon dioxide at a flow rate of 0.1 L.min^−1^ were used as the carrier gas stream. This constant gas flow was maintained across the locust antenna throughout the experiment of gold anoparticles delivery. Odorants were delivered atop of a dry air or an aerosol stream^25^,^26^. Briefly, hexanol (Sigma Aldrich, USA) was diluted in mineral oil to achieve 1% concentration by volume (v/v). A controlled volume of static headspace from odor bottles (0.1 L/min) were injected into the main flow system by using a pneumatic picopump (WPI Inc., USA; PV-820). The odor delivery was precisely timed and controlled by a custom designed Labview software. Hexanol pulses were delivered for 4 s duration and with a 60 s inter-trial interval.

To record the multi-unit spiking activity from the projection neurons, a 16-channel, 4 × 4 silicon probe (NeuroNexus, USA) was inserted into the superficial layer of the antennal lobe. Electrode channels were electroplated with gold to obtain impedances in the range of 200–300 kΩ. The raw extracellular signals were amplified using a 10 K gain (customizes 16-channel amplifier purchased from Biology Electronics Shop; Caltech, Pasadena, USA), and filtered between 0.3 to 6 KHz ranges. The amplified and filtered signals were acquired at 15 KHz sampling rate using a LabView data acquisition system (PCI-MIO-16E-4 DAQ cards; National Instruments, USA). To quantify the population of projection neuron (nuron that distinguish by a long axon extending from a cell body) activity, all recorded spikes from a single recording site was detected by thresholding the voltage response at 2.75 times the standard deviation (s.d.) of the baseline voltage fluctuations.

### Biodistribution of AuNPs in the olfactory pathway

To confirm the gold nanoparticle transport and accumulation in the brain, qualitative characterizations were performed by fluorescence and electron microscopy, whereas quantitative estimation was carried out by mass spectroscopy. Details of the each technique are described below.

### Fluorescence microscopy

Locust antennae were cut, the brain were excised, rinsed with DI water, and blotted dry on a filter paper (Whatman, USA; Grade 1). Whole mount brains and antennae were used for the examination of fluorescence using 10X and 40X objective lenses (LD LCI Plan-Apochromat, Carl Zesis, Germany) with green fluorescent filter (T660LPXR, Carl Zesis, Germany). Images were captured by AxioVision camera system (Carl Zesis, Germany).

### Electron microscopy

To verify the accumulation of AuNPs in the brain, TEM analysis was performed on the exposed brain slices. First, the exposed brains were immersed in 2.5% glutaraldehyde for 4 hours. Next, the brain was rinsed with 0.1 M phosphate buffer three times and subjected to secondary fixation using 2% OsO_4_ for 3 hours, followed by dehydration of the tissue through a series of ethanol washes (10% to 100% v/v). The brain was then embedded with a mixture of epoxy resin and propylene oxide as a transitional solvent, followed by resin infiltration. Finally, the resin was polymerized at 60 °C and the brain was ultra-sectioned with a thickness of 75 nm using an ultramicrotome (Leica Inc., USA). Sliced sections were then stained with uranyl acetate (4% v/v) for enhancing the electron micrograph contrast before imaging using TEM (FEI Inc., USA) at 120 KV, and the images were taken at various magnification (20000 to 97000 X).

### Elemental quantification by Inductive Coupled Plasma – Mass Spectrometry (ICP-MS)

The exposed and the control (unexposed) locust brains and antennae were individually digested in a mixture of 6 mL aqua regia (1:3 molar ratio of nitric acid: hydrochloric acid) and 1 mL hydrogen peroxide (30%, v/v) at 150 °C using microwave digestion (CEM MARS 6 Xpress, CEM Corp., USA). After complete digestion, each sample was suspended in 5 mL DI water and filtered through a 25 mm syringe filter with a 0.45 μm nylon membrane (VWR Inc., USA). These filtered samples were analyzed with the aid of an ELAN DRC II ICP-MS (Perkin Elmer, Inc., USA) to determine the concentration of elemental gold in each sample. Based on the raw data of elemental detection intensity, nanoparticles uptake and accumulation were calculated.

### Statistical analyses

All the sample measurements were performed in n = 3 or n = 10, and statistical analyses were performed using Microsoft Excel V.2013 software. Differences were considered significant when P-value was p < 0.01.

## Additional Information

**How to cite this article**: Raliya, R. *et al*. Non-invasive aerosol delivery and transport of gold nanoparticles to the brain. *Sci. Rep.* **7**, 44718; doi: 10.1038/srep44718 (2017).

**Publisher's note:** Springer Nature remains neutral with regard to jurisdictional claims in published maps and institutional affiliations.

## Supplementary Material

Supplementary Information
