# Supplementary material for: Non-invasive aerosol delivery and transport of gold nanoparticles to the brain
Source: Sci Rep. 2017 Mar 16;7:44718. doi: 10.1038/srep44718 (PMC5353651; doi:10.1038/srep44718)
Supplement: Supplementary Information [file srep44718-s1.pdf]

Supplementary data for

Non-invasive aerosol delivery and transport of gold nanoparticles to the brain

Ramesh Raliya[1], Debajit Saha[2], Tandeep S. Chadha[1], Baranidharan Raman[2] & Pratim Biswas[1,*]

[1] School of Engineering and Applied Science, Washington University in St Louis, St Louis, Missouri 63130, USA

[2] Systems Neuroscience and Neuromorphic Engineering Laboratory, Department of Biomedical Engineering, Washington University in St Louis, St Louis, Missouri 63130, USA

Table of Contents:

1. **Functionlization of AuNPs**
2. **Experimental plan**
3. **Calculation of AuNPs uptake efficiency**
4. **ICP-MS analysis of locust food and other controls**
5. **Fluroscence micrographs of locust brain and antenna (control experiments).**

1. Functionlization of AuNPs

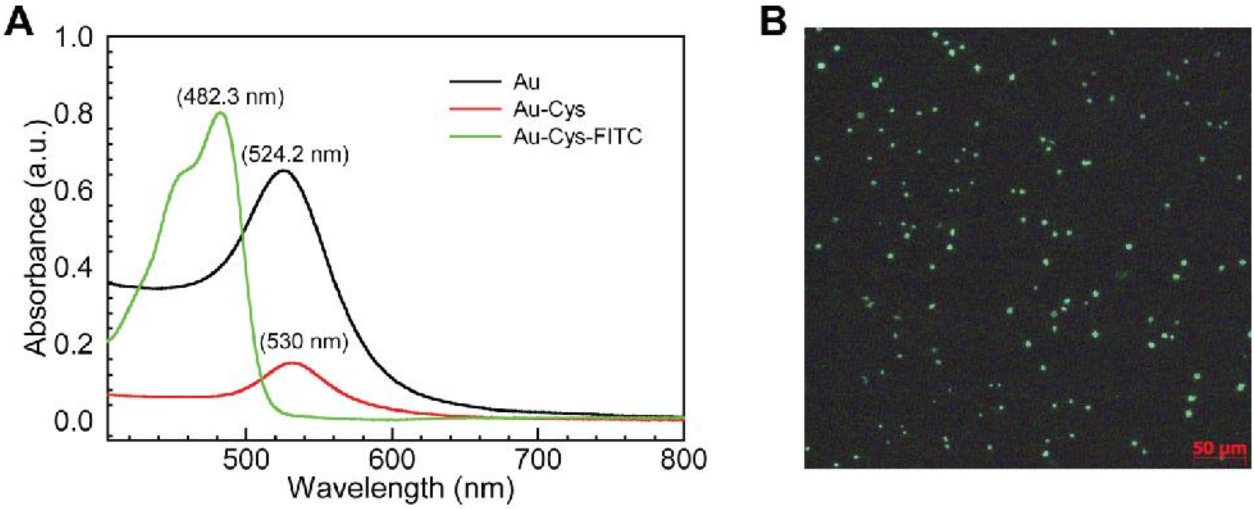

Figure S1. Functionlization of AuNPs

(A) Absorbance maxima of AuNPs at various sub stages of functionalization are shown. **(B)** Observed fluorescence of AuNPs under an optical microscope is shown. Nanoparticles are functionalized by fluorescein isothiocyanate and cysteine.

2. Characterization of functionalized AuNPs

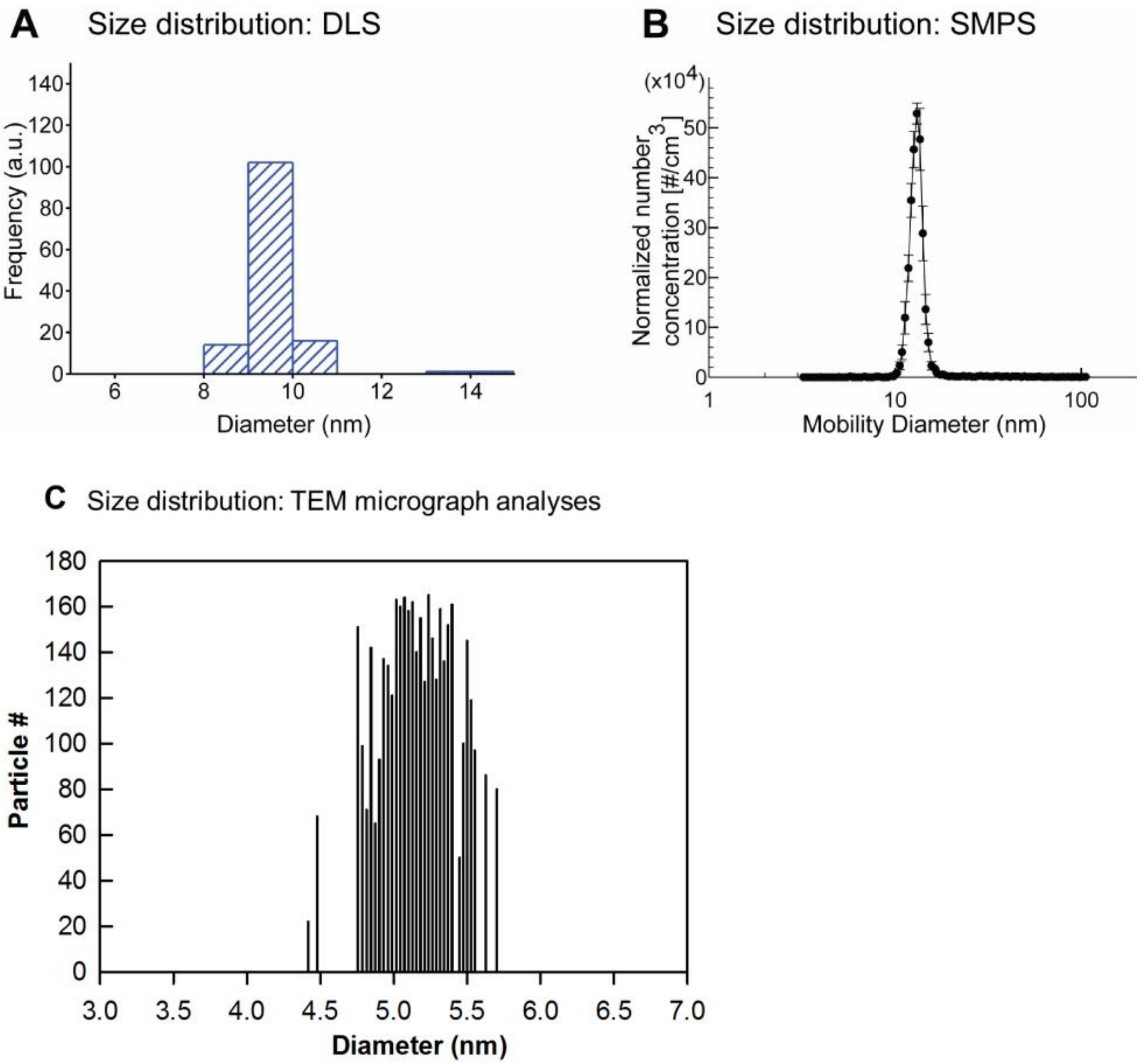

Figure S2. Characterization studies of functionalized AuNPs

(A) Hydrodynamic diameter and particle size distribution of nanoparticles in water are shown from Dynamic Light Scattering (DLS) technique. **(B)** Real time characterization of particle droplet size distribution during electrospray aerosol exposure is shown. The data was obtained by Scanning Mobility Particle Sizer (SMPS). **(C)** Size distribution of individual particles from TEM micrograph (inset) using ImageJ software.

3. AuNP Aerosol exposure experimental setup

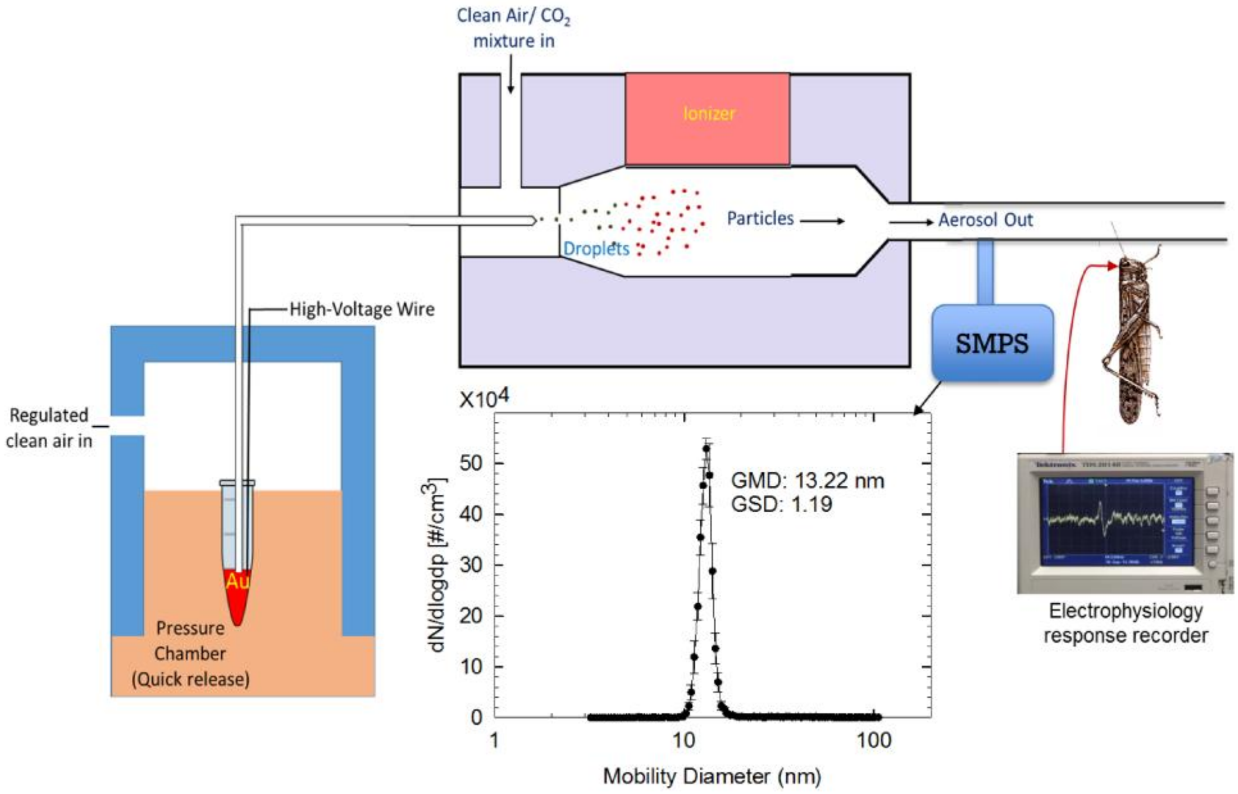

Figure S3. AuNP aerosol exposure experimental setup

An overall experimental plan of aerosol based nanoparticle delivery is shown. Gold nanoparticle aerosol was generated by electrospray chamber (TSI Inc.) and exposed to one of the locust antennae. Notice that only the part of the antenna inside the delivery tube gets exposed to the nanoparticles. Real time exposure concentration and mobility diameter were recorded by online particle measurement technique, scanning mobility particle sizer (SMPS).

4. Calculation of AuNPs uptake efficiency

Aerosol concentration measured by the SMPS: 1.59×10^5 #/cc

Transport efficiency with diffusive particle loss in the sampling line:

$$\eta_{tube,diff} = 1 - 2.56\xi^{\frac{2}{3}} + 1.2\xi + 0.177\xi^{\frac{4}{3}} \text{ for } \xi < 0.02$$

$$\eta_{tube,diff} = 0.819\exp(-3.657\xi) + 0.097\exp(-22.3\xi) + 0.032\exp(-57\xi) \text{ for } \xi > 0.02$$

Where $\xi = \frac{\pi DL}{Q_{sample}}$

$D = particle\ diffusion\ coefficient$; $L\ is\ the\ length\ of\ the\ sampling\ line$;
$Q_{sample}\ is\ the\ volumetric\ flow\ rate\ in\ the\ sampling\ line$

Using the above efficiency calculation, the number concentration of the particles in the delivery tube is estimated to be 2.34×10^5 #/cc. The mass concentration for the measured size distribution (after diffusion loss correction) was calculated as follows:

$$M(\mu g.cm^{-3}) = \sum_{d_p=3.22\ nm}^{d_p=105.5\ nm} \frac{\pi d_p^{\ 3}}{6}\rho_p N(d_p)$$

The mass delivery rate was estimated to be 0.316 $\mu g.h^{-1}$ by the following equation:
$m_{aerosol}\ (\mu g.h^{-1}) = M \times Q_{tot}$

Where ρ_p is the density of the gold nanoparticle (19.2 $g.cm^{-3}$), Q_{tot} is the total flow rate in the delivery tube (1.1 $L.min^{-1}$)

The nanoparticle uptake in the brain measured as a fraction of the mass in the aerosol flow, estimate as $m_{brain}/m_{aerosol}$ to be 0.00887

5. ICP-MS analyses of locust food and other controls

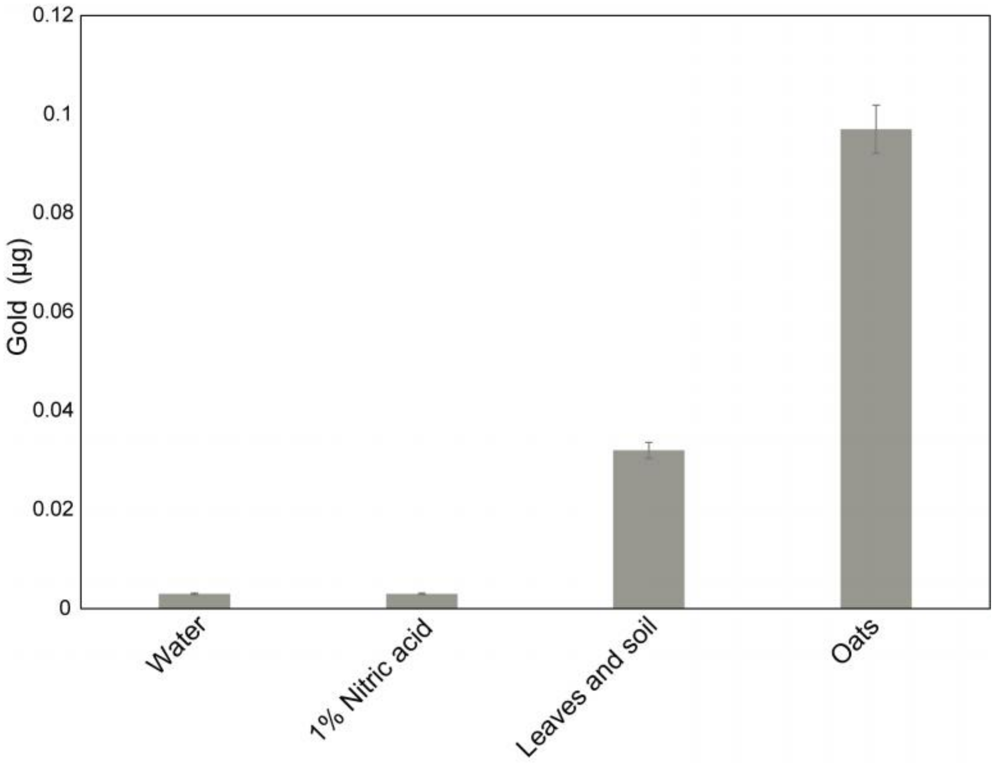

Figure S4. ICP-MS analysis of locust food and other controls

ICP-MS analysis of locust food (leaves and soil, oats) and other chemicals (nitric acid, DI water) used in ICP-MS experiments are shown for control purposes.

5. Fluroscence micrographs of locust brain and antenna.

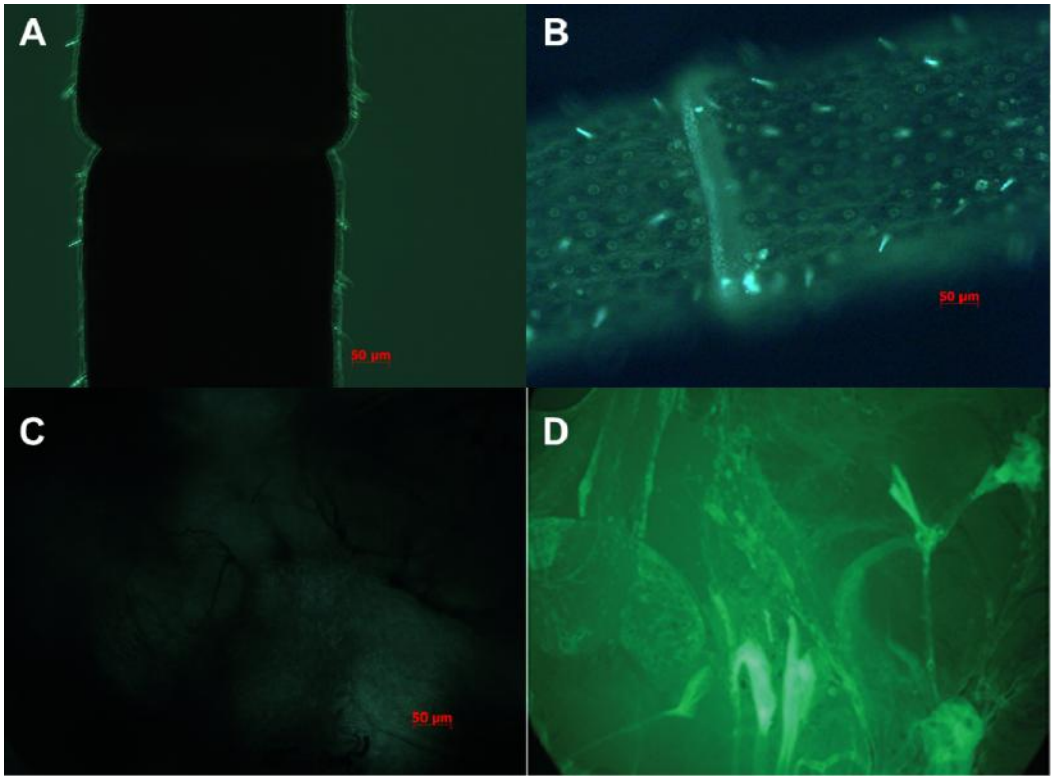

Figure S5. Fluroscence microscopy of locust brain and antenna. (A) Control antenna, **(B)**AuNPs exposed antenna, **(C)** Control brain and **(D)** AuNPs exposed brain. Scale bar for all images is 50 micrometer.
